# The cell cycle regulatory gene polymorphisms TP53 (rs1042522) and MDM2 (rs2279744) in lung cancer: a meta-analysis

**DOI:** 10.18699/VJ20.673

**Published:** 2020-11

**Authors:** O. Bulgakova, A. Kussainova, R. Bersimbaev

**Affiliations:** L.N. Gumilyov Eurasian National University, Institute of Cell Biology and Biotechnology, Nur-Sultan, Kazakhstan; L.N. Gumilyov Eurasian National University, Institute of Cell Biology and Biotechnology, Nur-Sultan, Kazakhstan; L.N. Gumilyov Eurasian National University, Institute of Cell Biology and Biotechnology, Nur-Sultan, Kazakhstan

**Keywords:** TP53 (rs1042522) and MDM2 (rs2279744) gene polymorphism, lung cancer, meta-analysis., полиморфизм генов TP53 (rs1042522) и MDM2 (rs2279744), рак легкого, метаанализ

## Abstract

Lung cancer is one of the most common types of cancer in the world. Although the mechanism of lung cancer is still unknown, a large number of studies have found a link between gene polymorphisms and the risk of lung cancer. The tumor suppressor p53 plays a crucial role in maintaining genomic stability and tumor prevention. MDM2 is a critical regulator of the p53 protein. Despite the importance of p53 pathway in cancer, data on the contribution of SNPs of TP53 (rs1042522) and MDM2 (rs2279744) to the development of lung cancer are very contradictory. A metaanalysis that collects quantitative data from individual studies and combines their results has the advantage of improving accuracy, providing reliable estimates, and resolving those issues in which studies on individual associations are not effective enough. The aim of this study was to determine whether the TP53 (rs1042522) and MDM2 (rs2279744) polymorphisms confer susceptibility to lung cancer. A meta-analysis was conducted on the associations between the TP53 (rs1042522) and MDM2 (rs2279744) polymorphisms and lung cancer. A total of 51 comparison studies including 25,366 patients and 25,239 controls were considered in this meta-analysis. The meta-analysis showed no association between lung cancer and MDM2 (rs2279744) under any model. A noteworthy association of TP53 (rs1042522) with susceptibility to lung cancer in overall pooled subjects was observed under three different models (allele contrast, homozygote contrast (additive) and dominant). Stratification by ethnicity indicated an association between the TP53 (rs1042522) and lung cancer in Asians and Caucasians. This meta-analysis demonstrates that the TP53 (rs1042522), but not MDM2 (rs2279744) polymorphism may confer susceptibility to lung cancer.

## Abstract (ru)

Рак легкого – один из наиболее распространенных видов рака в мире. Хотя механизм возникнове- ния заболевания по-прежнему остается в значительной степени неизвестным, благодаря многочисленным ис- следованиям была выявлена связь между полиморфизмами генов и риском развития рака легкого. Решающую роль в поддержании стабильности генома и профилактике опухолей играет онкосупрессор р53. Ключевым регулятором белка р53 является MDM2. Несмотря на важность p53 сигнального пути в канцерогенезе, данные о вкладе SNP TP53 (rs1042522) и MDM2 (rs2279744) в развитие рака легкого очень противоречивы. Метаанализ, собирающий количественные данные из отдельных исследований и объединяющий их результаты, имеет пре- имущество, которое заключается в повышении точности, предоставлении надежных оценок и решении тех вопросов, когда исследование отдельных ассоциаций недостаточно эффективно. Целью нашей работы было изучение роли полиморфизмов ТP53 (rs1042522) и MDM2 (rs2279744) в формировании предрасположенности к раку легкого. Проведен метаанализ ассоциации полиморфизмов TP53 (rs1042522) и MDM2 (rs2279744) и рака легкого. В общей сложности рассмотрено 51 исследование типа «случай–контроль», включающее 25 366 паци- ентов с раком легкого и 25 239 здоровых индивидуумов. Результаты метаанализа показали отсутствие связи между раком легкого и MDM2 (rs2279744) во всех моделях. Примечательно, что ассоциация TP53 (rs1042522) с предрасположенностью к раку легкого наблюдалась в трех разных моделях (мультипликативная, аддитивная и доминантная). Стратификация по этническому признаку также указывает на связь между TP53 (rs1042522) и риском развития рака легкого как в азиатской, так и в европейской популяции. Проведенный метаанализ по- зволяет сделать вывод, что полиморфизм ТР53 (rs1042522), но не MDM2 (rs2279744), может обусловливать пред- расположенность к раку легкого.

## Introduction

Lung cancer remains one of the most common forms of cancer in the world. Every year World Health Organization (WHO) includes lung cancer in the lists of the leading cause of death worldwide. Thus, there were 2.1 million cases of lung cancer and 1.8 million deaths in 2018 (https://www.who.int/ru/newsroom/fact-sheets/detail/the-top-10-causes-of-death). Cancer incidence rate varies in different regions of our planet, so the highest incidence of lung cancer is observed in Eastern Europe and Central and East Asia (Bray et al., 2018).

A large number of researches have been conducted to study the molecular base of lung cancer. One of the risk factors for the development of pulmonary neoplasms is genes polymorphisms. The main cause of carcinogenesis is disorders in the regulation of cell cycle control. The tumor suppressor gene TP53 plays an important role in regulating the cell cycle. p53 protein is known as the “guardian of the genome”. p53 regulates many genes expression in response to cellular stress induced by various adverse environmental factors (Haronikova et al., 2019). This protein plays a key role in processes such as DNA repair, cell cycle arrest, apoptosis and senescence (Nicolai et al., 2015). MDM2 is a key regulator of p53 protein activity and degradation. Polymorphic variants of the TP53 and MDM2 genes have been found in various types of cancer, including lung cancer. Analysis of the literature data showed that polymorphisms of the TP53 Arg72Pro (rs1042522) and MDM2 SNP309 (rs2279744) genes cause an increased predisposition to tumor development. The TP53 (rs1042522) gene polymorphism is localized on chromosome 17 position 7676154 Genotype frequency in the Caucasian population GG: 0.074, CC: 0.503, CG: 0.423. In the East Asian population, GG: 0.173, CC: 0.345, CG: 0.482 (http://www.ensembl.org/).

Meta-analyses have shown that the TP53 Arg72Pro polymorphic allele is associated with the development of stomach cancer (Xiang et al., 2012), bladder (Xu et al., 2012), colorectal cancer (Tian et al., 2017) and acute lymphocytic leukemia (Tian et al., 2016). However, no association was found between TP53 Arg72Pro and the risk of acute myeloid leukemia (Tian et al., 2016), oral squamous cell carcinoma (Sun et al., 2018), and esophagus cancer (Jiang et al., 2011).

The polymorphic allele of the MDM2 gene rs2279744 is located at 68808800 position on chromosome 12 Genotype frequency in the Caucasian population TT: 0.404, GG: 0.113, GT: 0.483. In the East Asian population TT: 0.200, GG: 0.276, GT: 0.524 (http://www.ensembl.org/).The MDM2 SNP309 polymorphism was also found to increase the risk of colorectal cancer (Qin et al., 2013), breast cancer (Cheng et al., 2012) and liver cancer (Tang et al., 2014). But there was no association with prostate, urinary tract (Ding et al., 2016) and stomach (Ma et al., 2013).

Many population studies have been conducted on the influence of the mutant alleles TP53 Arg72Pro (rs1042522) and MDM2 SNP309 (rs2279744) on the predisposition to the development of pulmonary neoplasia. It was shown that the polymorphism of the TP53 Arg72Pro gene is associated with a high risk of small cell lung cancer among Spaniards (Fernández-Rubio et al., 2008). Similar data were found for non-small cell lung cancer in Norwegians (Lind et al., 2007) and Poles (Szymanowska et al., 2006), squamous cell lung cancer in German residents (Popanda et al., 2007), and lung adenocarcinoma in the Chinese population (Zhang X. et al., 2006; Ren et al., 2013).

Data on the contribution of MDM2 SNP309 to the development of lung cancer are very contradictory. Most studies have shown an association of the MDM2 (rs2279744) mutant allele with a high risk of lung tissue carcinogenesis (Enokida et al., 2014; Wang X. et al., 2015; Li, 2017). However, Pine et al. (2006) did not find that MDM2 SNP309 is associated with lung neoplasia in the European population.

The data on the association of polymorphisms of the TP53 genes Arg72Pro (rs1042522) and MDM2 SNP309 (rs2279744) with the development of tumors as a whole are very contradictory. Therefore, it would be interesting to perform a metaanalysis on the association of TP53 Arg72Pro (rs1042522) and MDM2 SNP309 (rs2279744) with a risk of developing lung cancer in Asian and European populations.

## Materials and methods

**Search strategy.** Search for relevant studies was conducted using online databases, such as Scopus, PubMed and Web of Science. The search strategy was performed using a combination of the following keywords: “TP53”, “Murine double minute 2” or “MDM2”, “polymorphism”, “SNP”, “rs1042522”, “rs2279744”, “Arg72Pro”, “codon 72 Arg”, “c.215C > G”, “SNP309”, “c.291 T > G” “lung cancer”, “non-small cell lung cancer”, “association”.

**Inclusion and exclusion criteria.** The eligible inclusion criteria for the meta-analysis were (i) case-control study, (ii) identification of different histological types of lung cancer which was confirmed histologically or pathologically, (iii) having an available genotype for estimating an odds ratio (OR) with 95 % confidence interval (95 % CI), (iv) genotype frequencies in controls were consistent with those expected from Hardy–Weinberg equilibrium ( p > 0.05).

The studies were excluded when (i) they were not casecontrol studies, (ii) with duplicated data from previous articles, (iii) they were not original articles, e. g. review, (iv) inadequate genotype data were available.

**Data extraction and quality assessment.** Two researchers (O.B. and A.K.) evaluated the eligibility of all retrieved studies and extracted the pertinent data from the specified publications in standardized tables. The extracted data included: (i) the first author name, (ii) publication year, (iii) ethnicity, (iv) lung cancer patients and healthy controls sample size for each studied polymorphism. Disagreement was resolved by consulting with a third investigator (R.B.). The study quality was assessed in accordance with the Newcastle–Ottawa Scale (NOS) (Wells et al., 2009).

**Statistical analysis.** Hardy–Weinberg equilibrium (HWE) in control population was assessed utilizing the “Calculation of Chi-square test for deviation from Hardy–Weinberg equilibrium” online software (http://www.husdyr.kvl.dk/htm/kc/popgen/genetik/applets/kitest.htm). The statistical analysis was performed using Comprehensive Meta Analysis version 2.2.064 (Biosta, Englewood, NJ, USA). Estimates were summarized as ORs with 95 % CIs for each study. The heterogeneity was evaluated by using the I^2^ index. An I^2^ value of > 50 % was considered to indicate high heterogeneity (Lee, 2015). The random effects model for analysis was used in case high heterogeneity (Lee, 2015). Otherwise, the fixed-effects model was used. Publication bias was measured via “Begg’s funnel plot” and “Egger’s linear regression” method (Egger et al., 1997). A two-tailed p-value < 0.05 implied a statistically significant publication bias.

## Results

**Studies included in the meta-analysis**

A total of 531 potential articles were identified from the databases search. After 236 duplicate records were removed, a total of 295 potential articles were reviewed. Amongst these articles, 216 were excluded after titles and abstracts review. Afterwards, we excluded 28 studies for no case-control design. Finally, 51 studies with a total of 25,239 controls and 25,366 cases that met the inclusion criteria were included in this meta-analysis (Suppl. Fig. 1)^1^.

^1^Supplementary Materials are available in the online version of the paper: http://www.bionet.nsc.ru/vogis/download/pict-2020-24/appx13.pdf


**Characteristics of studies included in this meta-analysis**

A total of 37 articles that examined TP53 (rs1042522) association with lung cancer risk were determined. Two of these articles included data of two different sets (TP53 (rs1042522) and MDM2 (rs2279744)) (Zhang X. et al., 2006; Chua et al., 2010) and these sets were examined autonomously. Thus, the identified 37 articles encompassed case-controls studies involving 16,229 lung cancer patients and 14,897 controls (Table 1). Among 37 articles, 20 studies were established in Asian populations and 17 in Caucasian populations. The genotype frequencies in controls of all studies were consistent with those expected from HWE ( p > 0.05).

**Table 1.**
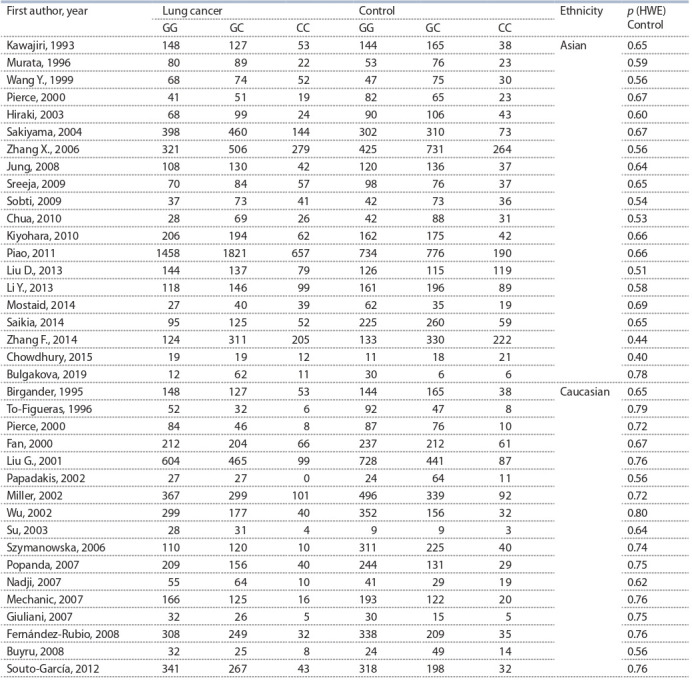
Characteristics of the studies of TP53 (rs1042522) polymorphism included in the meta-analysis

Another 14 articles identified MDM2 (rs2279744) association with increased lung cancer risk were retrieved (Table 2). These 14 articles encompassed case-controls studies involving 9,137 lung cancer patients and 10,342 controls. Among 14 articles, 7 studies were established in Asian populations and 7 in Caucasian populations. The genotype frequencies in controls of all studies were consistent with those expected from HWE ( p > 0.05).

**Table 2.**
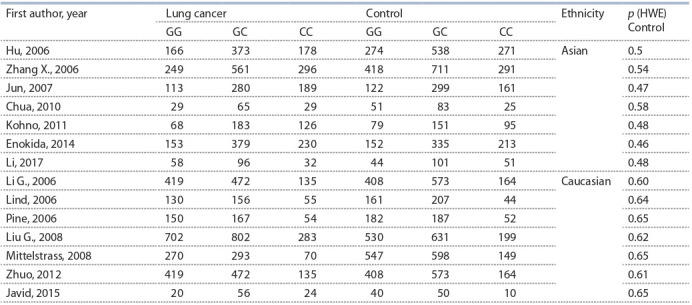
Characteristics of the studies of MDM2 (rs2279744) polymorphism included in the meta-analysis

All estimated published articles were executed under accredited genotyping methods.

**Meta-analysis of the relationship between the TP53 (rs1042522) polymorphism and lung cancer risk**

sociated with lung cancer (G versus C: OR = 0.82, 95 % CI 0.71–0.94, p = 0.005; GG versus CC: OR = 0.86, 95 % CI 0.74–0.99, p = 0.039; GG+GC versus CC: OR = 0.86, 95 % CI 0.76–0.98, p = 0.02; GG versus GC+CC: OR = 1.12, 95 % CI 0.89–1.42, p = 0.336). And the association was statistically significant under allele model (G versus C), homozygote model (GG versus CC) (Suppl. Fig. 2) and dominant model (GG+GC vs. CC) (Suppl. Fig. 3) ( p < 0.05). A summary of meta-analysis findings concerning associations between the TP53 (rs1042522) polymorphism and lung cancer risk is shown in Table 3.

**Table 3.**
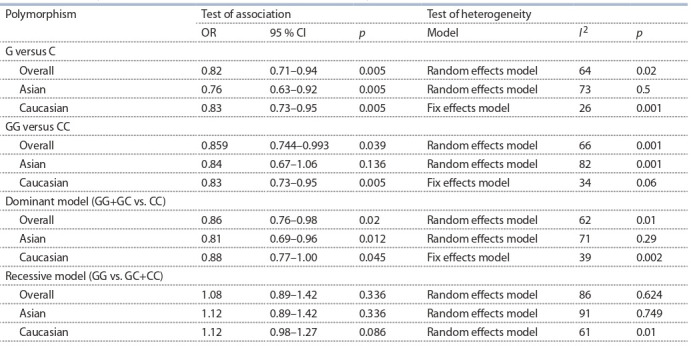
Meta-analysis of the association between TP53 (rs1042522) polymorphism and lung cancer risk

Further subgroup analysis was conducted on the association between TP53 (rs1042522) polymorphism and the risk of lung cancer (see Table 3). After stratifying by ethnicity, this metaanalysis indicated an obvious association of TP53 (rs1042522) and lung cancer risk among Caucasians (G versus C: OR = = 0.83, 95 % CI 0.73–0.95, p = 0.005; GG versus CC: OR = 0.83, 95 % CI 0.73–0.95, p = 0.005; GG+GC versus CC: OR = 0.88, 95 % CI 0.77–1.00, p = 0.045) and among Asians (G versus C: OR = 0.76, 95 % CI 0.63–0.92, p = 0.005; GG versus CC: OR = 0.84, 95 % CI 0.67–1.06, p = 0.136; GG+GC versus CC: OR = 0.81, 95 % CI 0.69–0.96, p = 0.012).

**Meta-analysis of the relationship between the MDM2 (rs2279744) polymorphism and lung cancer risk**

In this meta-analysis was shown no association MDM2 (rs2279744) polymorphism with lung cancer (G versus T: OR = 0.86, 95 % CI 0.71–1.03, p = 0.1; GG versus TT: OR = 0.86, 95 % CI 0.71–1.03, p = 0.1; GG+GT versus TT: OR = 0.90, 95 % CI 0.79–1.02, p = 0.5; GG versus GT+TT: OR = 1.10, 95 % CI 0.94–1.22, p = 0.276). A summary of meta-analysis findings concerning associations between the MDM2 (rs2279744) polymorphism and lung cancer risk is shown in Table 4. Subgroup analysis detected no association MDM2 (rs2279744) polymorphism with lung cancer.

****

Between-study heterogeneities were found in all subjects for both polymorphisms TP53 (rs1042522) and MDM2 (rs2279744) (see Table 3, 4). Because of this the meta-analysis was designed using “a random effect model” to establish pooled OR and corresponding 95 % CI for all models. We performed the meta-regression to explore the potential source of between-study. A big problem for meta-analysis is the disproportionate number of positive studies that leads to a bias in the publication. The funnel plot indicated some evidence of publication bias for Caucasians, but not for Asians in analysis of TP53 (rs1042522) and MDM2 (rs2279744) gene polymorphisms (Suppl. Fig. 4, 5). The publication bias was observed from Egger’s test ( p ≤ 0.05) also for Caucasian population (see Table 3, 4).

**Table 4.**
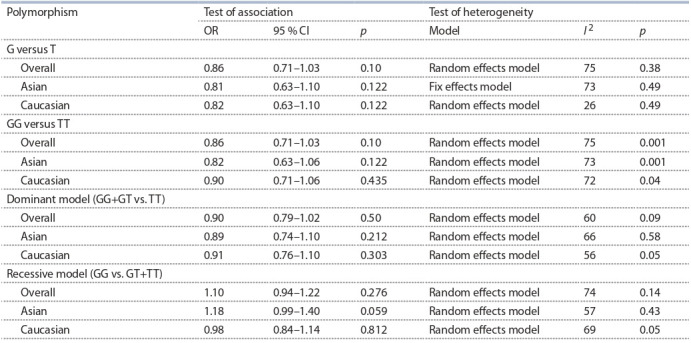
Meta-analysis of the association between MDM2 (rs2279744) polymorphism and lung cancer risk

## Discussion

The tumor suppressor gene TP53 (previously named p53), is key regulator of a cell cycle network, apoptosis and DNA repair pathway. TP53 is one of the most carcinogenesis-associated genes. There were several studies assessing the effects of TP53 polymorphisms on the risk of lung cancer, but the results are very contradictory. For example, no associations of the TP53 (rs1042522) polymorphism with lung cancer were found in Jung et al.’s (2008) article. But, increased risk to develop lung cancer was observed in association with the Pro/Pro genotype variant in Chowdhury et al.’s (2015) research. Mostaid et al. (2014) found that TP53 Arg72Pro and Pro72Pro genotype significantly associated with increased relative risk of lung cancer. Our previous study also demonstrated the association of genotype Arg72Pro of TP53 gene with lung cancer risk (Bulgakova et al., 2019). Papadakis et al. (2002) demonstrated that subjects with Arg72Arg genotype of rs1042522 had significantly increased lung cancer risk. We comprehensively searched the up-to-date electronic databases to reveal the associations between TP53 genetic polymorphisms (rs1042522) and risk of lung cancer. The genome-wide association study (GWAS) is very popular method to detect a variation in SNPs with variation in common diseases. In 2017, data from a study of new loci of susceptibility to lung cancer were published. The study identified RNASET2, SECISBP2L, NRG1, CHRNA2, OFBC1 and RTEL1 as candidate genes associated with lung cancer (McKay et al., 2017). The polymorphisms of TP53 (rs1042522) and MDM2 (rs2279744) weren’t detected in this GWAS (McKay et al., 2017).

A total of 37 case-control comparisons for TP53 (rs1042522) (16,229 lung cancer patients and 14,897 healthy controls) were investigated in this meta-analysis. A noteworthy association of TP53 (rs1042522) with susceptibility to lung cancer in overall pooled subjects was observed under three different models: the allele contrast, homozygote contrast (additive) and dominant model. Also, stratification analysis explained a strong evidence of this variant with risk of lung cancer among Asians and Caucasian under allelic, homozygote (only for Caucasian) and dominant models. Moreover, the Arg72Arg genotype was associated with the obvious protective effect (OR = 0.82, 95 % CI 0.71–0.94, p = 0.005).

Compared to TP53, whose role has been widely discussed in lung cancer developing, its main negative modifier – MDM2, has not been sufficiently studied. The data on the association of polymorphism of MDM2 (rs2279744 or 309T > G) with the risk of developing lung cancer as well as in the case of TP53 (rs1042522) are contradictory. Thus, Enokida et al. (2014) did not found any association between polymorphism of MDM2 (rs2279744) and lung cancer risk. Chua et al. (2010) demonstrated that the MDM2 (rs2279744) TT rather than the GG genotype is associated with increased risk of lung cancer in Asian. But, the MDM2 TT genotype was associated with a decreased risk of developing NSCLC compared with that of the MDM2 GG genotypes in Li G. et al.’s (2006) research. A total of 14 case-control comparisons for MDM2 (rs2279744) (9,137 lung cancer patients and 10,342 healthy controls) were investigated in this meta-analysis. There were no significant associations between MDM2 (rs2279744) polymorphisms and lung cancer with regard to G allele vs. T allele: OR = 0.86, 95 % CI 0.71–1.03, p = 0.1; homozygote model: OR = 0.86, 95 % CI 0.71–1.03, p = 0.1; dominant model: OR = 0.90, 95 % CI 0.79–1.02, p = 0.5 and recessive model: OR = 1.10, 95 % CI 0.94–1.22, p = 0.276. The stratification analysis also did not demonstrate the association of this polymorphism with risk of lung cancer among Asians and Caucasian under all models. Thus, MDM2 (rs2279744) polymorphism does not affect the risk of developing lung cancer.

This meta-analysis has some limitations. First, heterogeneity level was high. But we tried to eliminate this effect using a random effects model rather than a fixed effects model. Publication bias could also have biased the results, as studies that produced negative results may not have been published. Despite our use of Egger’s regression test, we cannot eliminate the possibility of bias. Second, the relative importance of the MDM2 (rs2279744) polymorphism during the development of lung cancer may vary between ethnic groups, but we were only able to perform ethnic-specific meta-analysis in Asians and Europeans. Thus, our results are applicable to only these ethnic groups. Therefore, additional studies with other ethnic populations are warranted to assess the association between MDM2 (rs2279744) polymorphism and the risk of lung cancer.

But, the present meta-analysis has also several strengths. We used a strong comprehensive search strategy, and had a well-defined inclusion and exclusion criteria. Reviewers performed the study selection and extracted data independently. Moreover, we assessed the quality of the included studies by predefined criteria and the score of included studies was high. Finally, all genotype data extracted from the studies were reported in the study. The advantage of this study over other meta-analyzes is a more complete review of literature and the inclusion of recent data.

## Conclusion

In summary, this meta-analysis study indicated evidence of association for TP53 (rs1042522), but not MDM2 (rs2279744) variants with lung cancer based on 51 case-control published studies. Additionally, stratified analysis based on ethnicity observed an obvious association of TP53 (rs1042522) both among Asian and European subjects under allelic, homozygote and dominant models. However, polymorphism MDM2 (rs2279744) may not impart susceptibility to lung cancer in either Asians or Europeans.

## Conflict of interest

The authors declare no conflict of interest.
